# Clinical response and on-treatment clinical remission with tezepelumab in a broad population of patients with severe, uncontrolled asthma: results over 2 years from the NAVIGATOR and DESTINATION studies

**DOI:** 10.1183/13993003.00316-2024

**Published:** 2024-12-05

**Authors:** Michael E. Wechsler, Guy Brusselle, J. Christian Virchow, Arnaud Bourdin, Konstantinos Kostikas, Jean-Pierre Llanos, Stephanie L. Roseti, Christopher S. Ambrose, Gillian Hunter, David J. Jackson, Mario Castro, Njira Lugogo, Ian D. Pavord, Neil Martin, Christopher E. Brightling

**Affiliations:** 1 Division of Pulmonary, Critical Care and Sleep Medicine, National Jewish Health, Denver, CO, USA; 2 Department of Respiratory Medicine, Ghent University Hospital, Ghent, Belgium; 3 Department of Pneumology and Department of Intensive Care Medicine, University of Rostock, Rostock, Germany; 4 PhyMedExp, University of Montpellier, CNRS, INSERM, CHU Montpellier, Montpellier, France; 5 Respiratory Medicine Department, University of Ioannina, Ioannina, Greece; 6 Global Medical Affairs, Amgen, Thousand Oaks, CA, USA; 7 Late-stage Development, Respiratory and Immunology, BioPharmaceuticals R&D, AstraZeneca, Gaithersburg, MD, USA; 8 Respiratory and Immunology, BioPharmaceuticals Medical, AstraZeneca, Gaithersburg, MD, USA; 9 Biometrics, Late-stage Development, Respiratory and Immunology, BioPharmaceuticals R&D, AstraZeneca, Cambridge, UK; 10 Guy's Severe Asthma Centre, Guy's and St Thomas’ NHS Foundation Trust, London, UK; 11 School of Immunology and Microbial Sciences, King's College London, London, UK; 12 Division of Pulmonary, Critical Care and Sleep Medicine, University of Kansas School of Medicine, Kansas City, KS, USA; 13 Department of Medicine, Division of Pulmonary and Critical Care Medicine, University of Michigan, Ann Arbor, MI, USA; 14 Respiratory Medicine, National Institute for Health and Care Research, Oxford Biomedical Research Centre, Nuffield Department of Medicine, University of Oxford, Oxford, UK; 15 Institute for Lung Health, National Institute for Health and Care Research, Leicester Biomedical Research Centre, University of Leicester, Leicester, UK; 16 Respiratory and Immunology, BioPharmaceuticals Medical, AstraZeneca, Cambridge, UK

## Abstract

**Background:**

In asthma, clinical response is characterised by disease improvement with treatment, whereas clinical remission is characterised by long-term disease stabilisation with or without ongoing treatment. The proportions of patients receiving tezepelumab who responded to treatment and who achieved on-treatment clinical remission were assessed in the NAVIGATOR (ClinicalTrials.gov identifier NCT03347279) and DESTINATION (ClinicalTrials.gov identifier NCT03706079) studies of severe, uncontrolled asthma.

**Methods:**

NAVIGATOR and DESTINATION were phase 3, randomised, double-blind, placebo-controlled studies; DESTINATION was an extension of NAVIGATOR. Complete clinical response was defined as achieving all of the following: ≥50% reduction in exacerbations *versus* the previous year, improvements in pre-bronchodilator (BD) forced expiratory volume in 1 s (FEV_1_) of ≥100 mL or ≥5%, improvements in Asthma Control Questionnaire (ACQ)-6 score of ≥0.5 and physician's assessment of asthma improvement. On-treatment clinical remission was defined as an ACQ-6 total score ≤1.5, stable lung function (pre-BD FEV_1_ >95% of baseline) and no exacerbations or use of oral corticosteroids during the time periods assessed.

**Results:**

Higher proportions of tezepelumab than placebo recipients achieved complete clinical response over weeks 0–52 (46% *versus* 24%; OR 2.83, 95% CI 2.10–3.82) and on-treatment clinical remission over weeks 0–52 (28.5% *versus* 21.9%; OR 1.44, 95% CI 0.95–2.19) and weeks >52–104 (33.5% *versus* 26.7%; OR 1.44, 95% CI 0.97–2.14). Tezepelumab recipients who achieved on-treatment clinical remission *versus* complete clinical response at week 52 had better preserved lung function and lower inflammatory biomarker levels at baseline, and fewer exacerbations in the 12 months before the study.

**Conclusions:**

Among patients with severe, uncontrolled asthma, tezepelumab treatment was associated with an increased likelihood of achieving complete clinical response and on-treatment clinical remission compared with placebo. Both are clinically important outcomes, but may be driven by different patient characteristics.

## Shareable abstract

**Tezepelumab treatment was associated with an increased likelihood of achieving a complete clinical response over 52 weeks as well as achieving on-treatment clinical remission over 2 years in patients with severe, uncontrolled asthma** https://bit.ly/3TiAkA7

## Graphical abstract

**Figure:**
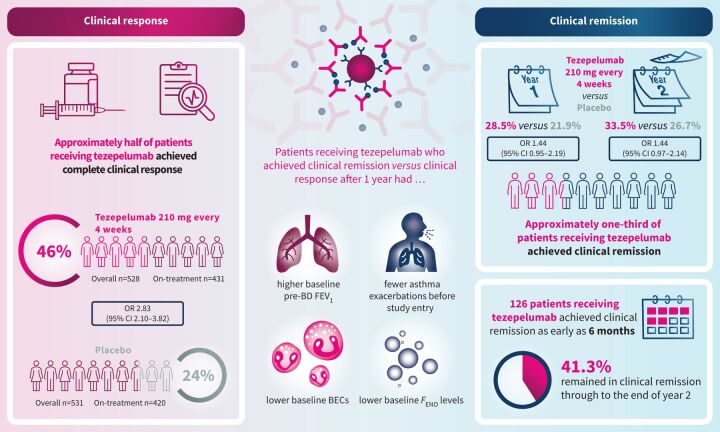
Tezepelumab treatment was associated with an increased likelihood of achieving a complete clinical response and on-treatment clinical remission *versus* placebo in patients with severe, uncontrolled asthma. Clinical response data are taken from the NAVIGATOR phase 3 trial (52 weeks). Clinical remission data are taken from the DESTINATION phase 3 trial (104 weeks). BD: bronchodilator; BEC: blood eosinophil count; *F*_ENO_: fractional exhaled nitric oxide; FEV_1_: forced expiratory volume in 1 s.

## Introduction

Globally, ∼262 million people are affected by asthma [1], resulting in a significant healthcare and economic burden [2]. Asthma is a heterogeneous, chronic lung disease that affects both adults and children [2]. Different underlying disease processes give rise to multiple clinical asthma phenotypes, thereby contributing to the complexity of treatment strategies, particularly because many patients with asthma have overlapping phenotypes (*e.g.* both eosinophilic and allergic) [3].

Severe asthma may be defined as asthma that remains uncontrolled despite treatment with high-dose inhaled corticosteroids (ICS) plus a long-acting β_2_-agonist (with or without oral corticosteroids (OCS)) and treatment of contributory factors, or as asthma that worsens when high-dose treatment is decreased [4, 5]. Patients with severe asthma may be prescribed biologic therapies as adjuncts to reduce exacerbations, reduce OCS exposure and improve disease control [5]. Tezepelumab targets thymic stromal lymphopoietin (TSLP), an epithelial cytokine that has multifaceted effects on the initiation and persistence of inflammation in asthma [6]. Inhibition of TSLP by tezepelumab is associated with reduced numbers of occlusive mucus plugs and reduced airway hyperresponsiveness, probably due to effects on eosinophils, mucus production, mast cells and airway smooth muscle cells [6–9]. Tezepelumab is approved for the treatment of severe asthma without phenotypic restrictions [10, 11]. In the phase 3 NAVIGATOR study (ClinicalTrials.gov identifier NCT03347279), tezepelumab reduced the annualised asthma exacerbation rate and improved lung function, asthma control and health-related quality of life (HRQoL) compared with placebo in patients with severe, uncontrolled asthma [12]. Patients who completed treatment in NAVIGATOR could enrol into the DESTINATION long-term extension study (ClinicalTrials.gov identifier NCT03706079). In DESTINATION, tezepelumab treatment was well tolerated for up to 2 years and, consistent with the NAVIGATOR study, resulted in sustained, clinically meaningful reductions in asthma exacerbations, with improved lung function, asthma control and HRQoL [13].

In recent years, the treatment goals for severe asthma have shifted from clinical response to clinical remission and, ultimately, to disease modification. Clinical response is typically based on achieving improvements surpassing minimum clinically important differences (MCIDs) in single outcome measures of symptoms, asthma control or HRQoL [14]. However, definitions are not standardised [15]. Some patients with severe asthma achieve a good response to biologics which can be predicted to some extent by their baseline inflammatory profile [14, 16], but may never achieve disease remission. It is anticipated that earlier intervention with potentially disease-modifying therapies such as biologics will provide long-term benefits to patients with severe asthma [17, 18].

Remission now appears to be a feasible treatment goal in asthma, having been successfully implemented as a goal in other chronic inflammatory conditions [18–20]. Currently, there are no standard, globally accepted criteria for asthma remission; however, the concept has been incorporated into several national guidelines for asthma [21]. Proposed criteria for achieving clinical remission from asthma societies and consensus groups generally include ≥12 months with no significant symptoms, no exacerbations, stable and optimised lung function and no use of systemic corticosteroids [17, 22–30], although some recent studies have also assessed remission both with and without using a lung function criterion [31–33]. Patients meeting some but not all criteria achieve partial clinical remission. Clinical remission definitions may be used for patients on treatment or off treatment, but definitions for patients off treatment typically also require no asthma treatment for ≥12 months [22]. Complete remission requires both clinical remission and normalisation of the underlying pathology (*i.e.* biological remission), indicated by low levels of inflammatory biomarkers and, if appropriate, negative bronchial hyperresponsiveness [22].

This study assessed the effect of tezepelumab on clinical response (in NAVIGATOR) and on-treatment clinical remission (in DESTINATION) in patients with severe, uncontrolled asthma.

## Methods

### Study design

NAVIGATOR and DESTINATION were phase 3, multicentre, randomised, double-blind, placebo-controlled, parallel-group studies of patients with severe, uncontrolled asthma. Patients who were randomised to receive tezepelumab 210 mg every 4 weeks for 52 weeks in NAVIGATOR continued receiving this dosing regimen for 52 weeks in DESTINATION, whereas those who received placebo in NAVIGATOR were re-randomised 1:1 to receive either tezepelumab 210 mg every 4 weeks or placebo in DESTINATION [13].

### Participants

Patients (aged 12–80 years) in NAVIGATOR had physician-diagnosed asthma and had been receiving medium- or high-dose ICS (fluticasone propionate ≥500 µg·day^−1^ or equivalent) for ≥12 months before screening and at least one additional controller medication, with or without OCS, for ≥3 months before the date of informed consent [12].

Patients must have had an Asthma Control Questionnaire (ACQ)-6 score of ≥1.5 and at least two asthma exacerbations in the previous year that led to hospitalisation or an emergency department visit that resulted in systemic corticosteroid treatment. A broad population of adults and adolescents were enrolled in NAVIGATOR, including those with baseline blood eosinophil counts (BECs) of <300 cells·μL^−1^ (58.4% of patients) and ≥300 cells·μL^−1^ (41.6% of patients). Full details of the study design and inclusion and exclusion criteria have been published previously [12, 13].

The study was conducted in accordance with the ethical principles of the Declaration of Helsinki, International Council for Harmonisation good clinical practice guidelines, and applicable regulatory requirements. Approvals from local independent ethics committees were obtained, and all patients or their legal guardians provided written informed consent in accordance with local requirements.

### Outcomes

**FIGURE 1:**
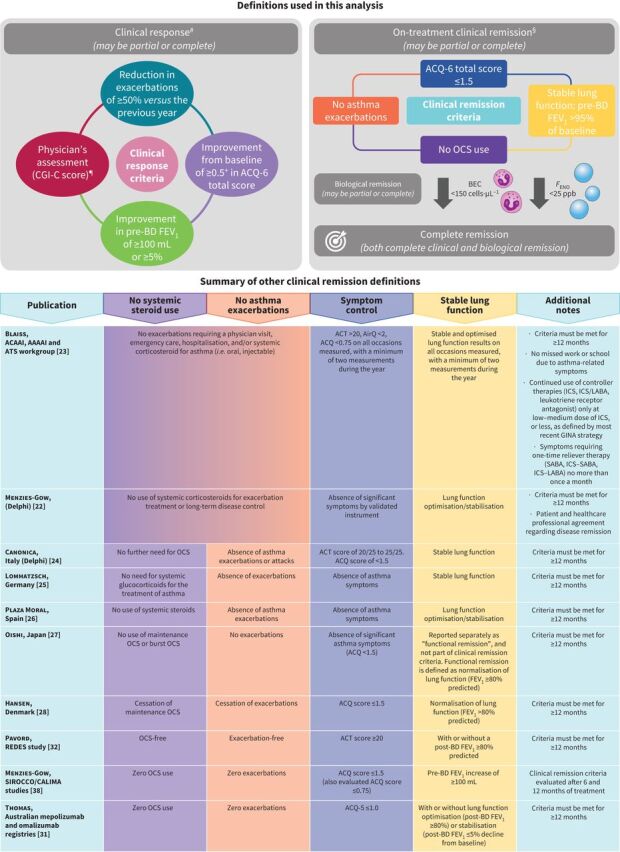
Clinical response and clinical remission definitions used in this analysis and summary of clinical remission definitions from the literature. CGI-C: Clinical Global Impression of Change; ACQ: Asthma Control Questionnaire; BD: bronchodilator; FEV_1_: forced expiratory volume in 1 s; OCS: oral corticosteroid; BEC: blood eosinophil count; *F*_ENO_: fractional exhaled nitric oxide; ACAAI: American College of Allergy, Asthma and Immunology; AAAAI: American Academy of Allergy, Asthma, and Immunology; ATS: American Thoracic Society; ACT: Asthma Control Test; AirQ: Asthma Impairment and Risk Questionnaire; ICS: inhaled corticosteroid; LABA: long-acting β_2_-agonist; GINA: Global Initiative for Asthma; SABA: short-acting β_2_-agonist. ^#^: clinical response was measured at week 52 of NAVIGATOR; ^¶^: physician's assessment of improvement was expressed as a CGI-C score and was defined as minimally improved, much improved or very much improved; ^+^: the minimum clinically important difference in ACQ-6 score is 0.5; ^§^: on-treatment clinical remission was measured across multiple time points over the 2 years of DESTINATION.

As part of a pre-specified exploratory analysis, the proportions of partial and complete clinical responders in NAVIGATOR over 52 weeks were assessed. The proportions of clinical responders were evaluated for each of the four clinical response criteria assessed: a reduction in exacerbations of ≥50% *versus* the previous year; physician's assessment of improvement, expressed as a Clinical Global Impression of Change score (minimally improved, much improved or very much improved); improvement from baseline of ≥100 mL (the MCID) or ≥5% in pre-bronchodilator (BD) forced expiratory volume in 1 s (FEV_1_); and improvement from baseline of ≥0.5 (the MCID) in ACQ-6 score. Partial clinical responders were defined as those who met between one and three of these criteria, and complete clinical responders were defined as those meeting all four criteria in this analysis (figure 1).

As part of a *post hoc* analysis, the proportions of patients in NAVIGATOR who achieved on-treatment clinical remission in DESTINATION were assessed over the following time points: weeks 0–24, weeks >24–52, weeks 0–52 and weeks >52–104. On-treatment clinical remission was defined as meeting all of the following criteria (figure 1): ACQ-6 total score ≤1.5 (controlled asthma) at the end of the period assessed; a pre-BD FEV_1_ of >95% of the baseline level at the end of the period assessed; no use of OCS during the period assessed; and no asthma exacerbations during the period assessed. Spirometry assessments in clinical practice have inherent variability and two FEV_1_ measurements within 5% of each other are generally considered to be an acceptably reproducible result [34]. Therefore, for the lung function criterion, a pre-BD FEV_1_ of >95% of the baseline value was selected for this analysis to be confident that a patient's lung function was stable from baseline to the end of the treatment period and that it had not declined. Additional details and end-points related to clinical remission can be found in the supplementary methods.

### Statistical analyses

Clinical response and on-treatment clinical remission rates were compared between treatment groups using logistic regression models, with treatment, region and age group as covariates. Full details of the statistical analyses are provided in the supplementary methods.

## Results

**FIGURE 2:**
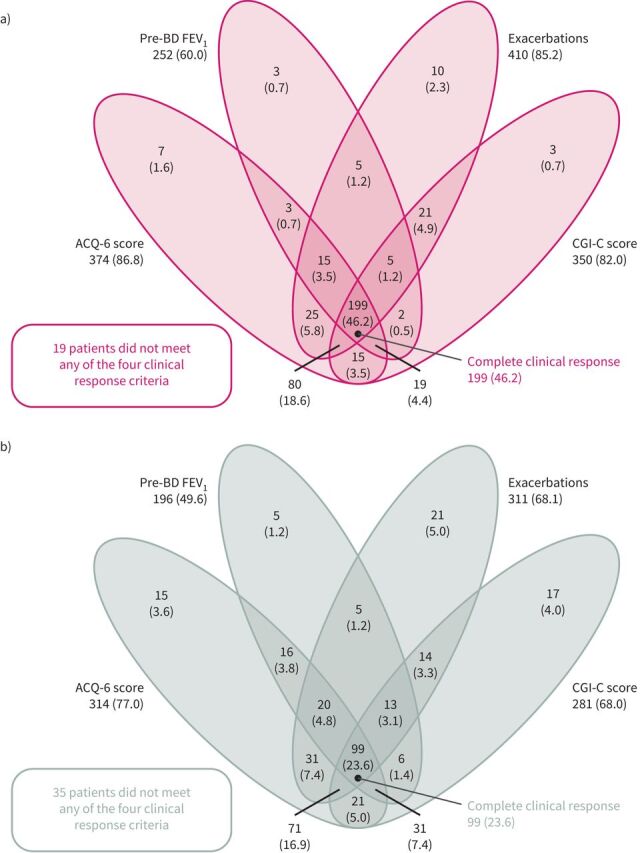
The proportion of patients receiving a) tezepelumab 210 mg every 4 weeks and b) placebo with individual and overlapping components of clinical response at week 52. Results are reported as n (%). ACQ: Asthma Control Questionnaire; BD: bronchodilator; FEV_1_: forced expiratory volume in 1 s; CGI-C: Clinical Global Impression of Change.

Overall, in NAVIGATOR, 528 patients received tezepelumab and 531 received placebo, of whom 431 and 420, respectively, both completed the on-treatment period and had data available to assess clinical response criteria. Across the individual clinical response criteria, there were higher proportions of responders in the tezepelumab group than in the placebo group (figure 2 and supplementary figure S1). Compared with placebo, a higher proportion of patients who received tezepelumab achieved a complete clinical response (*i.e.* met all four response criteria) over 52 weeks (24% *versus* 46%, respectively (figure 2); OR 2.83, 95% CI 2.10–3.82 (supplementary figure S1)). There were 19 (4.0%) and 35 (7.6%) patients in the tezepelumab and placebo groups, respectively, who did not meet any of the four clinical response criteria (nonresponders). Baseline demographics and clinical characteristics were generally similar between on-treatment complete and partial/nonresponder subgroups (supplementary table S1). Compared with placebo, a greater proportion of complete clinical responders in the tezepelumab group were receiving maintenance OCS at baseline, had more than two exacerbations in the 12 months before the study, had higher baseline BECs and had higher baseline fractional exhaled nitric oxide (*F*_ENO_) levels.

**FIGURE 3:**
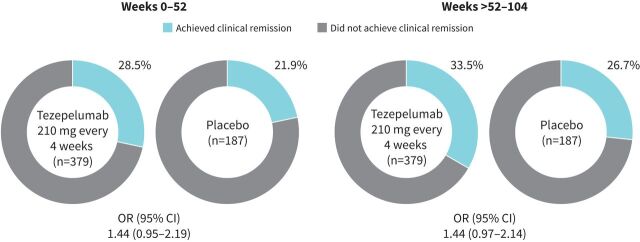
The proportion of patients receiving tezepelumab 210 mg every 4 weeks or placebo who achieved on-treatment clinical remission during weeks 0–52 and weeks >52–104. In this analysis, for patients who completed treatment with data missing at week 104, the next available off-treatment measurement was input at week 104 for the Asthma Control Questionnaire-6 and pre-bronchodilator forced expiratory volume in 1 s criteria. An OR of >1 favours tezepelumab. There was one patient receiving tezepelumab who stopped oral corticosteroid use in DESTINATION and did achieve on-treatment clinical remission at week 52.

**FIGURE 4:**
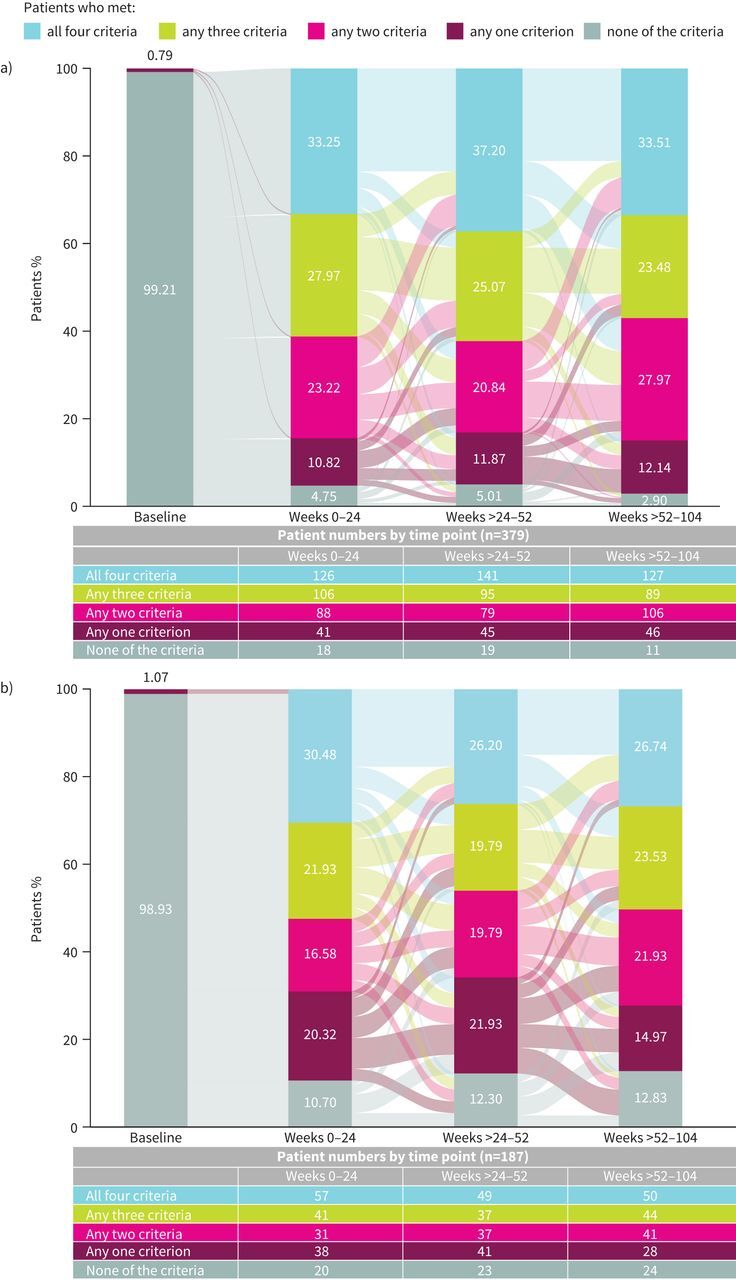
The proportion of patients receiving a) tezepelumab 210 mg every 4 weeks and b) placebo who achieved on-treatment clinical remission criteria over weeks 0–24, >24–52 and >52–104. In this analysis, for patients who completed treatment with data missing at week 104, the next available off-treatment measurement was input at week 104 for the Asthma Control Questionnaire (ACQ)-6 and pre-bronchodilator forced expiratory volume in 1 s criteria. Blue shading indicates the proportion of patients over weeks 0–24, >24–52 and >52–104 who met all four remission criteria since the previous period. At baseline, the purple shading represents patients who met both the study inclusion criterion and the remission criterion for ACQ-6 score (*i.e.* a score of ≤1.5). There was one patient receiving tezepelumab who stopped oral corticosteroid use in DESTINATION and did achieve on-treatment clinical remission at week 52.

Of the patients included in the on-treatment clinical remission analysis, the baseline demographics and clinical characteristics were generally well balanced between those receiving tezepelumab (n=379) and placebo (n=187) (supplementary table S2). Over weeks 0–52 (year 1), 28.5% of patients receiving tezepelumab and 21.9% of patients receiving placebo achieved on-treatment clinical remission (OR 1.44, 95% CI 0.95–2.19) (figure 3). Over weeks >52–104 (year 2), 33.5% of patients receiving tezepelumab and 26.7% of patients receiving placebo achieved on-treatment clinical remission (OR 1.44, 95% CI 0.97–2.14) (figure 3). The proportion of patients receiving tezepelumab *versus* placebo who achieved on-treatment clinical remission was 33.3% *versus* 30.5%, respectively, over weeks 0–24 (OR 1.15, 95% CI 0.79–1.69), and 37.2% *versus* 26.2%, respectively, over weeks >24–52 (OR 1.69, 95% CI 1.14–2.50) (figure 4). Findings over weeks >52–104 where patients with missing data at week 104 were assumed not to have achieved clinical remission (supplementary figure S2) were similar to those presented in figures 3 and 4. Experiencing an exacerbation was the most frequent reason why clinical remission was not achieved over year 1 or year 2 among patients receiving tezepelumab (supplementary figure S3; data for placebo are shown in supplementary figure S4).

**FIGURE 5:**
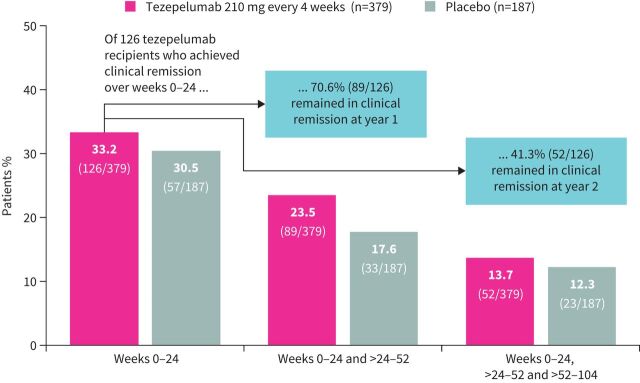
The proportions of patients receiving tezepelumab 210 mg every 4 weeks or placebo who achieved clinical remission in weeks 0–24, and then remained in clinical remission throughout the on-treatment period. In this analysis, for patients who completed treatment with data missing at week 104, the next available off-treatment measurement was input at week 104 for the Asthma Control Questionnaire-6 and pre-bronchodilator forced expiratory volume in 1 s criteria. There was one patient receiving tezepelumab who stopped oral corticosteroid use in DESTINATION and did achieve on-treatment clinical remission at week 52.

The proportions of patients who achieved on-treatment clinical remission over weeks 0–24 and remained in clinical remission throughout the on-treatment period are shown in figure 5.

Among patients receiving tezepelumab, those who achieved on-treatment clinical remission over weeks 0–24, >24–52 and >52–104 had a higher baseline pre-BD FEV_1_ and higher baseline BECs and *F*_ENO_ levels than those who did not achieve on-treatment clinical remission over these time points (table 1; data for placebo are shown in supplementary table S3). Tezepelumab treatment was associated with a reduction in baseline BECs and *F*_ENO_ levels from baseline to week 104, in patients regardless of on-treatment clinical remission status over weeks >52–104, indicated by an increase in the proportion of patients with a BEC of <150 cells·μL^−1^ and a *F*_ENO_ level of <25 ppb (table 2).

**TABLE 1:** Baseline demographics and clinical characteristics for patients receiving tezepelumab 210 mg every 4 weeks who achieved and did not achieve clinical remission over weeks 0–24, >24–52 and >52–104^#^

	Achieved remission			Did not achieve remission		
	0–24 weeks	>24–52 weeks	>52–104 weeks^¶^	0–24 weeks	>24–52 weeks	>52–104 weeks^¶^
**Patients**	126	141	127	244	228	247
**Age years**	48.4±17.3	48.9±17.3	49.0±17.9	50.1±16.0	50.1±15.5	50.2±15.3
**Female**	72 (57.1)	81 (57.4)	76 (59.8)	160 (65.6)	151 (66.2)	158 (64.0)
**BMI kg·m^−2^**	27.9±6.1	28.4±6.3	28.1±6.3	29.6±7.8	29.5±7.8	29.5±7.6
**ICS dose group** ^+^						
Medium	39 (31.0)	38 (27.0)	26 (20.5)	53 (21.7)	51 (22.4)	66 (26.7)
High	87 (69.0)	103 (73.0)	101 (79.5)	191 (78.3)	177 (77.6)	181 (73.3)
**Pre-BD FEV_1_ L**	1.98±0.68	1.94±0.69	1.92±0.70	1.76±0.69	1.77±0.70	1.78±0.70
**Pre-BD FEV_1_ % predicted**	64.8±16.4	64.9±16.7	64.9±16.4	61.2±17.6	60.8±17.5	60.8±17.5
**FEV_1_ reversibility %**	15.6±15.2	15.5±14.9	16.2±16.4	13.7±13.8	13.9±14.3	13.9±13.5
**Age at asthma onset years**	27.9±20.2	26.0±20.1	26.4±19.5	24.8±19.2	25.8±19.1	25.9±19.6
**Duration of disease years**						
<20	9.5±4.8	9.4±5.1	9.9±4.8	9.6±5.2	9.8±5.1	9.4±5.2
≥20	34.9±12.6	36.0±11.6	36.1±12.1	39.3±12.4	39.4±12.9	38.9±12.7
**Duration of disease years**						
<20	73 (57.9)	71 (50.4)	67 (52.8)	117 (48.0)	118 (51.8)	124 (50.2)
≥20	53 (42.1)	70 (49.6)	60 (47.2)	127 (52.0)	110 (48.2)	123 (49.8)
**Exacerbations in the 12 months before enrolment in NAVIGATOR**						
2	78 (61.9)	92 (65.2)	79 (62.2)	150 (61.5)	139 (61.0)	154 (62.3)
>2	48 (38.1)	49 (34.8)	48 (37.8)	94 (38.5)	89 (39.0)	93 (37.7)
***F*_ENO_ level ppb**						
Mean±sd	42.9±35.5	43.2±35.7	42.0±34.0	38.9±33.7	37.5±32.5	38.6±33.7
Median (min–max)	33.5 (5.0–213.0)	33.0 (5.0–213.0)	33.0 (6.0–173.0)	29.0 (5.0–198.0)	27.5 (5.0–198.0)	28.0 (5.0–213.0)
***F*_ENO_ group ppb**						
<25	48 (38.7)	52 (37.1)	49 (39.8)	108 (45.0)	104 (46.4)	109 (44.5)
≥25 to <50	35 (28.2)	43 (30.7)	35 (28.5)	66 (27.5)	61 (27.2)	70 (28.6)
≥50	41 (33.1)	45 (32.1)	39 (31.7)	66 (27.5)	59 (26.3)	66 (26.9)
**BEC cells·µL^−1^**						
Mean±sd	380±399	366±370	348±373	296±235	291±242	309±256
Median (min–max)	295 (0–3650)	300 (0–3650)	280 (0–3650)	235 (10–1680)	220 (10–1680)	240 (0–1680)
**BEC group cells·µL^−1^**						
<150	24 (19.0)	28 (19.9)	28 (22.0)	67 (27.5)	64 (28.1)	65 (26.3)
150 to <300	39 (31.0)	42 (29.8)	39 (30.7)	87 (35.7)	85 (37.3)	89 (36.0)
<300	63 (50.0)	70 (49.6)	67 (52.8)	154 (63.1)	149 (65.4)	154 (62.3)
≥300	63 (50.0)	71 (50.4)	60 (47.2)	90 (36.9)	79 (34.6)	93 (37.7)
300 to <450	30 (23.8)	31 (22.0)	32 (25.2)	44 (18.0)	42 (18.4)	42 (17.0)
≥450	33 (26.2)	40 (28.4)	28 (22.0)	46 (18.9)	37 (16.2)	51 (20.6)
**Serum total IgE IU·mL^−1^**						
Mean±sd	518.1±833.9	557.7±1207.7	508.3±695.2	537.7±1093.6	512.6±871.0	540.4±1136.3
Median (min–max)	225.9 (1.5–6412.4)	239.0 (1.5–12 823.2)	235.5 (1.5–3664.7)	193.6 (1.5–12 823.2)	194.0 (1.5–6412.4)	194.4 (1.5–12 823.2)
**FEIA positive for any perennial aeroallergen^§^**	81 (64.3)	90 (63.8)	78 (61.4)	154 (63.1)	145 (63.6)	160 (64.8)
**Nasal polyps**	29 (23.0)	27 (19.1)	25 (19.7)	42 (17.2)	44 (19.3)	47 (19.0)

Data are presented as n, mean±sd or n (%), unless otherwise stated. BMI: body mass index; ICS: inhaled corticosteroid; BD: bronchodilator; FEV_1_: forced expiratory volume in 1 s; *F*_ENO­_: fractional exhaled nitric oxide; BEC: blood eosinophil count; Ig: immunoglobulin; FEIA: fluorescence enzyme immunoassay. ^#^: there was one patient receiving tezepelumab who stopped oral corticosteroid use in DESTINATION and did achieve on-treatment clinical remission at week 52; ^¶^: in this analysis, for patients who completed treatment with data missing at week 104, the next available off-treatment measurement was input at week 104 for the Asthma Control Questionnaire-6 and pre-BD FEV_1_ criteria; ^+^: medium-dose ICS: fluticasone propionate 500 µg·day^−1^ or equivalent; high-dose ICS: fluticasone propionate >500 µg·day^−1^ or equivalent; ^§^: positive for at least one perennial aeroallergen (cat dander, dog dander, cockroach, dust mite (*Dermatophagoides farinae*, *D. pteronyssinus*) and mould mix).

**TABLE 2:** Proportions of patients who achieved and did not achieve clinical remission at week 104 with low or high levels of inflammatory biomarkers at time points from baseline to week 104

	Achieved remission at week 104^#^	Did not achieve remission at week 104^#^
**Tezepelumab 210 mg every 4 weeks**	127	247
BEC <150 cells·μL^−1^ and *F*_ENO_ <25 ppb		
Baseline	14 (11.0)	45 (18.2)
Week 24	33 (26.0)	65 (26.3)
Week 52	42 (33.1)	93 (37.7)
Week 104	33 (26.0)	78 (31.6)
BEC ≥150 cells·μL^−1^ and *F*_ENO_ ≥25 ppb		
Baseline	61 (48.0)	116 (47.0)
Week 24	26 (20.5)	45 (18.2)
Week 52	19 (15.0)	36 (14.6)
Week 104	19 (15.0)	29 (11.7)
**Placebo**	50	132
BEC <150 cells·μL^−1^ and *F*_ENO_ <25 ppb		
Baseline	10 (20.0)	18 (13.7)
Week 24	6 (12.0)	21 (15.9)
Week 52	9 (18.0)	21 (15.9)
Week 104	8 (16.0)	20 (15.2)
BEC ≥150 cells·μL^−1^ and *F*_ENO­_ ≥25 ppb		
Baseline	22 (44.0)	51 (38.6)
Week 24	17 (34.0)	57 (43.2)
Week 52	15 (30.0)	44 (33.3)
Week 104	13 (26.0)	38 (28.8)

Data are presented as n or n (%). BEC: blood eosinophil count; *F*_ENO­_: fractional exhaled nitric oxide. ^#^: in this analysis, for patients who completed treatment with data missing at week 104, the next available off-treatment measurement was input at week 104 for the Asthma Control Questionnaire-6 and pre-bronchodilator forced expiratory volume in 1 s criteria.

The baseline clinical characteristics of patients receiving tezepelumab who achieved complete clinical response *versus* those who achieved on-treatment clinical remission at week 52 were evaluated (table 3). Patients who achieved on-treatment clinical remission had a higher baseline pre-BD FEV_1_, fewer exacerbations in the 12 months before the study, lower baseline BECs and lower baseline *F*_ENO­_ levels than those who achieved a complete clinical response.

**TABLE 3:** Baseline demographics and clinical characteristics for patients receiving tezepelumab 210 mg every 4 weeks who achieved complete clinical response *versus* those who achieved clinical remission over weeks 0–52^#^

	Achieved complete clinical response	Achieved on-treatment clinical remission
**Patients**	199	108
**Age years**	48.3±16.9	48.3±17.6
**Female**	119 (59.8)	58 (53.7)
**BMI kg·m^−2^**	28±6.3	28±6.1
**ICS dose group^¶^**		
Medium	45 (22.6)	29 (26.9)
High	154 (77.4)	79 (73.1)
**Maintenance OCS use**	18 (9.0)	0 (0.0)
**Pre-BD FEV_1_ L**	1.80±0.69	2.03±0.70
**Pre-BD FEV_1_ % predicted**	60.6±17.5	66.3±16.6
**Exacerbations in the 12 months before enrolment in NAVIGATOR**		
2	109 (54.8)	71 (65.7)
>2	90 (45.2)	37 (34.3)
***F*_ENO_ level ppb**		
Mean±sd	47.7±38.9	43.7±36.4
Median (min–max)	35.0 (5.0–213.0)	34.0 (5.0–213.0)
***F*_ENO_ group ppb**		
<25	65 (32.8)	37 (34.6)
≥25 to <50	61 (30.8)	35 (32.7)
≥50	72 (36.4)	35 (32.7)
**BEC cells·μL^−1^**		
Mean±sd	406±363	375±412
Median (min–max)	340 (20–3650)	290 (0–3650)
**BEC group cells·μL^−1^**		
<150	35 (17.6)	22 (20.4)
150 to <300	53 (26.6)	34 (31.5)
<300	88 (44.2)	56 (51.9)
≥300	111 (55.8)	52 (48.1)
300 to <450	44 (22.1)	21 (19.4)
≥450	67 (33.7)	31 (28.7)
**Serum total IgE IU·mL^−1^**		
Mean±sd	514.4±756.4	591.9±1339.7
Median (min–max)	206.1 (1.5–4357.4)	237.4 (1.5–12 823.2)
**FEIA positive for any perennial aeroallergen^+^**	116 (58.3)	69 (63.9)
**Nasal polyps**	43 (21.6)	23 (21.3)

Data are presented as n, mean±sd or n (%), unless otherwise stated. BMI: body mass index; ICS: inhaled corticosteroid; OCS: oral corticosteroid; BD: bronchodilator; FEV_1_: forced expiratory volume in 1 s; *F*_ENO­_: fractional exhaled nitric oxide; BEC: blood eosinophil count; Ig: immunoglobulin; FEIA: fluorescence enzyme immunoassay. ^#^: there was one patient receiving tezepelumab who stopped OCS use in DESTINATION and did achieve on-treatment clinical remission at week 52; ^¶^: medium-dose ICS: fluticasone propionate 500 µg·day^−1^ or equivalent; high-dose ICS: fluticasone propionate >500 µg·day^−1^ or equivalent; there was one patient in the placebo group who received fluticasone propionate <500 µg·day^−1^ or equivalent; ^+^: positive for at least one perennial aeroallergen (cat dander, dog dander, cockroach, dust mite (*Dermatophagoides farinae*, *D. pteronyssinus*) and mould mix).

## Discussion

In this analysis of patients with severe, uncontrolled asthma, tezepelumab treatment was associated with an increased likelihood of achieving complete clinical response over 52 weeks compared with placebo. Additionally, tezepelumab was associated with an increased likelihood of achieving and sustaining on-treatment clinical remission over 52 weeks and 104 weeks compared with placebo.

Almost half of tezepelumab recipients achieved a complete clinical response, compared with one-quarter of placebo recipients. The magnitude of clinical response to tezepelumab appeared to be greater in patients with more severe and uncontrolled disease and elevated inflammation at baseline. This was indicated by a higher proportion of complete responders in the tezepelumab group *versus* the placebo group having received maintenance OCS at baseline and experiencing more than two exacerbations in the past 12 months, as well as having higher baseline BECs and *F*_ENO_ levels.

Approximately one-third of patients receiving tezepelumab achieved on-treatment clinical remission at the time points assessed. Across all time points assessed, clinical remission among tezepelumab recipients was associated with higher BECs and *F*_ENO_ levels at baseline, suggesting that these characteristics may help to predict patients more likely to achieve clinical remission with tezepelumab. BECs and *F*_ENO_ levels were reduced with tezepelumab treatment *versus* placebo from baseline to week 104, irrespective of whether the patients achieved on-treatment clinical remission at week 104; this indicates that low levels of type 2 inflammation achieved after treatment do not necessarily predict clinical remission. It is yet to be fully understood how biomarker changes following TSLP blockade with tezepelumab relate to clinical response and remission, including whether biomarker reductions are required for clinical remission.

Interestingly, the odds of achieving a complete clinical response to treatment with tezepelumab in this analysis were higher than the odds of achieving on-treatment clinical remission. This may be because the patients included in NAVIGATOR and DESTINATION had long-term, severe, uncontrolled asthma. As response to treatment is a comparison to a patient's relative baseline status, patients with the most severe and uncontrolled underlying disease often demonstrate the largest response to biologic therapy. However, large clinical responses may not always translate to long-term disease stability. Remission is restoration to a state of relative wellbeing, which is best achieved by patients with less severe disease at baseline. Consequently, those with the most severe disease may never achieve disease remission, which is a limitation of clinical response as an asthma outcome. Additional consideration should be given to the presence of comorbidities or other conditions, such as obesity, when evaluating clinical response and clinical remission, because these factors may influence symptom control and HRQoL scores (*e.g.* ACQ-6) in patients with asthma [35].

It should be noted that a considerable number of placebo recipients in this study achieved clinical remission. Strong clinical improvements in patients receiving placebo have been observed previously in randomised controlled trials (RCTs) of biologics [36], probably because of optimisation of patients’ inhaler technique and patient education regarding ICS adherence, as well as the specialised care and monitoring that is provided by asthma experts during the trials.

Other RCTs of biologics have evaluated clinical remission using criteria similar to those used in this analysis [37], although differences in disease characteristics between patient populations should be considered when comparing remission rates. In the phase 3 SIROCCO and CALIMA studies in patients with severe eosinophilic asthma, 23.9% (140 out of 586) of patients not receiving OCS at baseline who received benralizumab and 14.8% (92 out of 620) of patients who received placebo achieved clinical remission at 12 months. This was defined as zero exacerbations, zero OCS use, an ACQ-6 score of ≤1.5 (≤0.75 was also evaluated) and a pre-BD FEV_1_ increase of ≥100 mL after 12 months. Using equivalent criteria at 6 months, 26.3% (160 out of 609) of benralizumab recipients and 19.0% (122 out of 643) of placebo recipients met remission criteria [38]. In the phase 3 QUEST study in patients with uncontrolled, moderate-to-severe, type 2 inflammatory asthma (as defined by a BEC of ≥150 cells·μL^−1^ or a *F*_ENO_ level of ≥20 ppb), 35.0% (364 out of 1040) of dupilumab recipients and 20.4% (111 out of 544) of placebo recipients achieved clinical remission at 12 months. This was defined as no exacerbations, no OCS use, an ACQ-5 score <1.5 and either an improvement in pre-BD FEV_1_ of ≥100 mL or a post-BD FEV_1_ of ≥80% predicted [39]. Furthermore, in the open-label TRAVERSE study, 36.1% of dupilumab recipients achieved clinical remission after an additional year of treatment [39]. The patient population evaluated in the QUEST and TRAVERSE studies of dupilumab included ∼50% of patients on medium-dose ICS who were consequently classified as having uncontrolled, moderate asthma. It is expected that these patients would be more likely to achieve clinical remission than clinical trial participants with severe, uncontrolled asthma, because those with moderate asthma have less severe disease at baseline.

Clinical remission with biologics has also been assessed in real-world observational studies using similar definitions. In the REDES study in patients with severe eosinophilic asthma who were newly prescribed mepolizumab in Spain, on-treatment clinical remission was defined as no OCS use, no exacerbations and an Asthma Control Test score of ≥20, with or without a post-BD FEV_1_ of ≥80% predicted normal. When the lung function component was included in the remission criteria, 30% of patients achieved clinical remission [32]. Lastly, in an Australian study of patients with severe asthma, clinical remission at 12 months was defined as zero exacerbations and zero OCS use during the previous 6 months and an ACQ-5 score of ≤1, with or without a post-BD FEV_1_ of either ≥80%, or ≤5% decline from baseline. When the lung function component was included in the remission criteria, 25.2% and 19.1% receiving mepolizumab and omalizumab, respectively, achieved clinical remission [31]. In summary, although definitions of clinical remission consistently use the criteria of “no exacerbations” and “no OCS use”, there is variation in how adequate symptom control is defined and in the inclusion of a lung function criterion, including whether lung function should be stable or improved (and how these are defined).

With improvements in asthma therapies in recent years, including the introduction of personalised, potentially disease-modifying biologic therapies, treatment goals have evolved from short-term control of symptoms and exacerbations to disease remission, a more meaningful long-term goal for the patient. In contrast to clinical remission, clinical response is influenced by baseline inflammation and disease severity before treatment is received, which was observed in our results. Therefore, treating patients sooner after diagnosis, before the disease progresses, could help to achieve remission; however, this needs to be assessed in appropriately designed RCTs [29]. Globally accepted criteria used to define clinical remission, biological remission and complete remission could assist in the design of such trials. Given the notable proportion of patients in the present study who achieved remission at 6 months and remained in remission at 12 months, we suggest that the 6-month time point should be considered by asthma societies and consensus groups developing definitions of remission. It is in the interest of patients and clinicians to know whether patients are achieving clinical remission earlier than 12 months into receiving a biologic therapy, because this may allow for earlier reduction in background medication use. Accordingly, the ARRIVAL study plans to assess the potential for patients receiving tezepelumab to reduce maintenance inhaled asthma therapy and will evaluate remission at 6 months.

There was a notable proportion of patients in the present study with severe asthma for ≥20 years, and this is likely to have impacted the proportion of patients achieving on-treatment clinical remission in this analysis. The concept of earlier intervention with biologic therapy to increase the chances of achieving remission before asthma becomes too severe or uncontrolled has been comprehensively explored in a longitudinal analysis using data from the International Severe Asthma Registry [40]. This should be explored in future clinical trials of biologics; the ARRIVAL study of tezepelumab will recruit patients with a shorter duration of asthma than in the present study and this will provide valuable insights into earlier biologic intervention, particularly for those with severe disease.

Strengths of this analysis include the robustness of the DESTINATION dataset, based on the large number of patients who continued from NAVIGATOR into DESTINATION. Additionally, the duration of DESTINATION allowed remission criteria to be assessed from 6 months to 2 years, and the effect of tezepelumab on remission could be evaluated *versus* placebo in year 2, which, to date, is an outcome not previously evaluated in RCTs of biologics in severe asthma. Further investigation into off-treatment clinical remission will be evaluated in the DESTINATION study.

A limitation of this analysis is the impact of the coronavirus disease 2019 pandemic, which coincided with year 2 of the study (*i.e.* DESTINATION). The pandemic may have influenced exacerbation rates, lung function and symptom control metrics because of the reduced risk of viral infections due to increased isolation arising from lockdown measures. Indeed, it was notable that exacerbations in both treatment groups decreased from year 1 to year 2 [12, 13]. In addition, the pandemic contributed to increased missing data at week 104. To evaluate potential bias from these missing data, a sensitivity analysis of the remission analyses was performed, which assumed nonremission for patients in instances where data were missing (rather than using imputed data); the findings were similar between the two analyses.

### Conclusion

Tezepelumab treatment was associated with an increased likelihood of achieving a complete clinical response over 52 weeks as well as achieving on-treatment clinical remission over 2 years in patients with severe, uncontrolled asthma. Clinical response as an asthma outcome is more likely to occur in those with high baseline inflammation and severe, uncontrolled disease; therefore, as a treatment goal, it has less relevance for the long-term needs of the patient. In contrast, clinical remission and complete remission in asthma appear to be much more meaningful goals. Further exploration of how baseline characteristics may help to predict remission, and how biomarker changes relate to response and remission, would be valuable to understand how patients may best benefit from biologic treatment.

## Supplementary material

10.1183/13993003.00316-2024.Supp1 **Please note:** supplementary material is not edited by the Editorial Office, and is uploaded as it has been supplied by the author.
Supplementary material ERJ-00316-2024.Supplement

## Shareable PDF

10.1183/13993003.00316-2024.Shareable1 This PDF extract can be shared freely online.
Shareable PDF ERJ-00316-2024.Shareable

## Data Availability

Data underlying the findings described in this manuscript may be obtained in accordance with AstraZeneca's data-sharing policy described at https://astrazenecagrouptrials.pharmacm.com/ST/Submission/Disclosure.
