# Supplementary material for: Clinical response and on-treatment clinical remission with tezepelumab in a broad population of patients with severe, uncontrolled asthma: results over 2 years from the NAVIGATOR and DESTINATION studies
Source: Eur Respir J. 2024 Dec 5;64(6):2400316. doi: 10.1183/13993003.00316-2024 (PMC11618813; doi:10.1183/13993003.00316-2024)
Supplement: Supplementary file 1 [file ERJ-00316-2024.Supplement.pdf]

Supplementary appendix

Clinical response and on-treatment clinical remission with tezepelumab in a broad population of patients with severe, uncontrolled asthma: results over 2 years from the NAVIGATOR and DESTINATION studies

Michael E. Wechsler[1], Guy Brusselle[2], J. Christian Virchow[3], Arnaud Bourdin[4], Konstantinos Kostikas[5], Jean-Pierre Llanos[6], Stephanie L. Roseti[7], Christopher S. Ambrose[8], Gillian Hunter[9], David J. Jackson[10,11], Mario Castro[12], Njira Lugogo[13], Ian D. Pavord[14], Neil Martin[15,16] and Christopher E. Brightling[15]

[1]National Jewish Health, Denver, CO, USA. [2]Department of Respiratory Medicine, Ghent University Hospital, Ghent, Belgium. [3]Department of Pneumology and Department of Intensive Care Medicine, University of Rostock, Rostock, Germany. [4]PhyMedExp, University of Montpellier, CNRS, INSERM, CHU Montpellier, Montpellier, France. [5]Respiratory Medicine Department, University of Ioannina, Ioannina, Greece. [6]Global Medical Affairs, Amgen, Thousand Oaks, CA, USA. [7]Late-stage Development, Respiratory and Immunology, BioPharmaceuticals R&D, AstraZeneca, Gaithersburg, MD, USA. [8]Respiratory and Immunology, BioPharmaceuticals Medical, AstraZeneca, Gaithersburg, MD, USA. [9]Biometrics, Late-stage Development, Respiratory and Immunology, BioPharmaceuticals R&D, AstraZeneca, Cambridge, UK. [10]Guy's Severe Asthma Centre, Guy's and St Thomas' NHS Foundation Trust, London, UK. [11]School of Immunology & Microbial Sciences, King's College London, London, UK. [12]Division of Pulmonary, Critical Care and Sleep Medicine, University of Kansas School of Medicine, Kansas City, KS, USA. [13]Department of Medicine, Division of Pulmonary and Critical Care Medicine, University of Michigan, Ann Arbor, MI, USA. [14]Respiratory Medicine, National Institute for Health and Care Research, Oxford Biomedical Research Centre, Nuffield Department of Medicine, University of Oxford, Oxford, UK. [15]Institute for Lung Health, National Institute for Health and Care Research, Leicester Biomedical Research Centre, University of Leicester, Leicester, UK. [16]Respiratory and Immunology, BioPharmaceuticals Medical, AstraZeneca, Cambridge, UK.

Corresponding author: Prof Christopher E Brightling

Email: ceb17@leicester.ac.uk

Tel: +44 116 250 2704

Address: Institute for Lung Health, National Institute for Health and Care Research, Leicester Biomedical Research Centre, University of Leicester, Leicester, UK.

Supplementary methods

Outcomes

The number of patients receiving tezepelumab or placebo who met specific combinations of the four remission criteria was assessed over weeks 0–52 and weeks >52–104. Furthermore, the proportion of patients who achieved on-treatment clinical remission over weeks 0–24 and then remained in clinical remission over weeks >24–52 and subsequently over weeks >52–104 was determined. Finally, the association of biomarker changes with clinical remission was assessed in patients who did and did not achieve on-treatment clinical remission at week 104. At multiple timepoints from baseline to week 104, the proportions of patients with both a blood eosinophil count (BEC) of <150 cells/μL and a fractional exhaled nitric oxide (FeNO) level of <25 ppb (i.e., evaluation of potential complete remission) and the proportion of patients with a BEC of ≥150 cells/μL and a FeNO level of ≥25 ppb were determined (time period analysed: weeks >52–104).

Statistical analyses

The on-treatment patient population was assessed at week 52 in NAVIGATOR for the clinical response analysis. A patient was considered to have completed the on-treatment period when the difference between the randomization date and the following dates (whichever was sooner) was greater than 359 days: date of the last dose of tezepelumab + 33 days, date of death, date of study withdrawal or last date when exacerbation status was known. Patients were excluded from the analysis if they did not complete the on-treatment period or had missing data at week 52 within any criterion. The odds ratios (ORs) and 95% confidence intervals (CIs) were estimated to compare treatment groups using a logistic regression model with treatment, region and age group as covariates.

Patients who received tezepelumab in both NAVIGATOR and DESTINATION or placebo in both NAVIGATOR and DESTINATION were included in the on-treatment clinical remission analysis; patients who received placebo in the parent study and were re-randomized to tezepelumab in DESTINATION were excluded from the analysis. Patients who completed treatment in the SOURCE study could also enrol in DESTINATION; however, these patients are not included in the present analysis because they were an OCS-dependent population during SOURCE and consequently could not be considered, per the clinical remission criteria during year 1. A small proportion of patients in NAVIGATOR, who were included in this analysis, were receiving maintenance OCS and were mandated to continue receiving these as part of the NAVIGATOR study design; therefore, by definition, these patients could not achieve on-treatment clinical remission. To circumvent any missing data at week 104 because of the coronavirus disease 2019 pandemic during the second year of

DESTINATION, patients who completed treatment with data missing for ACQ-6 score or pre-BD FEV_1 at week 104 were re-analysed using the next available off-treatment measurement. Safety or extended follow-up data from weeks 110, 116, 122 and 134 were used to replace missing ACQ-6 and pre-BD FEV_1 values at week 104. For completeness, a supplemental analysis of the proportion of patients who achieved clinical remission over weeks 0–24, >24–52 and >52–104 was performed where patients who completed treatment with data missing for ACQ-6 score or pre-BD FEV_1 at week 104 were assumed to have not achieved on-treatment clinical remission (i.e. patients missing at least one remission criterion measurement were considered not to be in clinical remission, even if they met all other remission criteria). ORs and 95% CIs were estimated using the same logistic regression model as the original analysis.

FIGURE S1 Proportion of NAVIGATOR patients meeting clinical response criteria at week 52 in the on-treatment population.

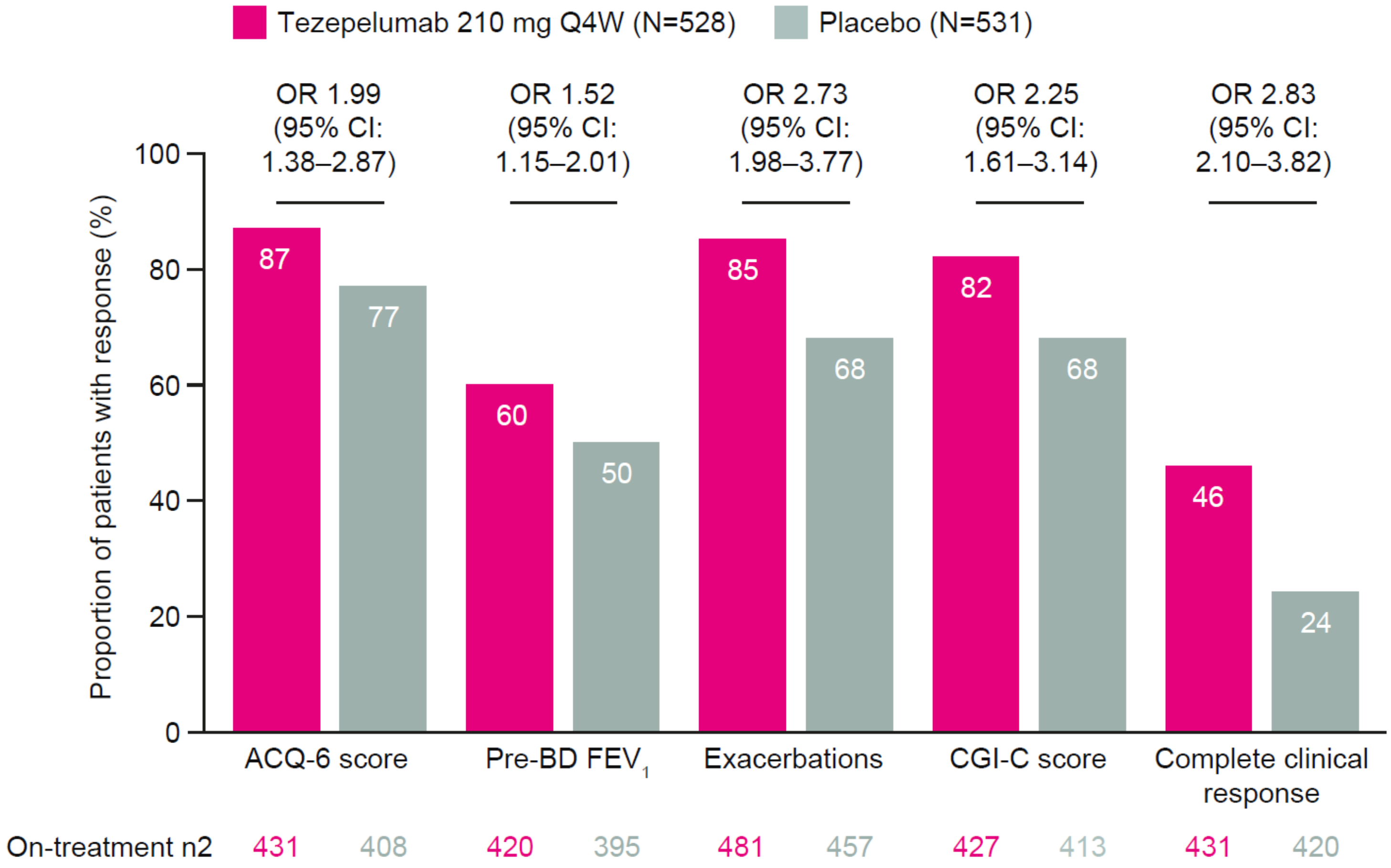

N denotes the number of patients in the full analysis set; on-treatment n2 denotes the number of patients in the treatment group who completed the on-treatment period and had non-missing on-treatment data available at week 52. Patients who completed the on-treatment period and had data available to assess response criteria were included in the responder analysis.

The OR (95% CI) for the intent-to-treat population (sensitivity analysis) at week 52 was 2.76 (2.09–3.65), in the complete clinical response group.

ACQ-6: Asthma Control Questionnaire-6; BD: bronchodilator; CGI-C: Clinical Global Impression of Change; CI: confidence interval: FEV_1: forced expiratory volume in 1 second; OR: odds ratio; Q4W: every 4 weeks.

FIGURE S2 The proportion of patients receiving tezepelumab a) or placebo b) who achieved on-treatment clinical remission over weeks 0–24, >24–52 and >52–104, where non-remission was assumed if data were missing at week 104.

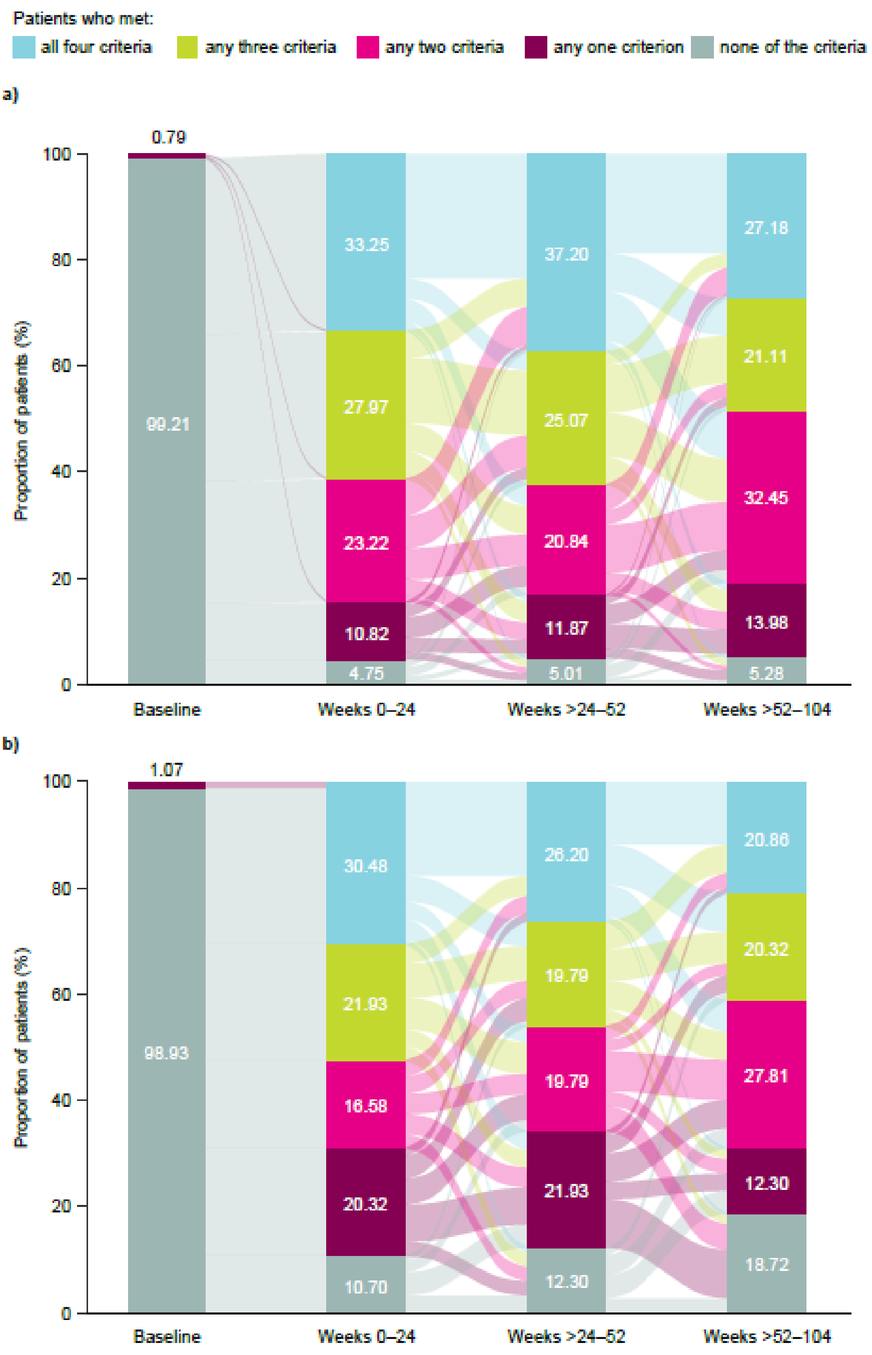

The number of patients meeting remission criteria over weeks >52 to 104, where non-remission was assumed if data were missing at week 104#		
	Tezepelumab 210 mg Q4W (N=379)	Placebo (N=187)
All four criteria	103	39
Any three criteria	80	38
Any two criteria	123	52
Any one criterion	53	23
None of the criteria	20	35

#This sensitivity analysis assumes that patients did not achieve clinical remission if data for ACQ-6 or pre-BD FEV_1 at week 104 were missing.

Blue shading indicates the proportion of patients over weeks 0–24, >24–52 and >52–104 who met all four remission criteria since the previous period. At baseline, the purple shading represents patients who met both the study inclusion criterion and the remission criterion for ACQ-6 score (i.e. a score of 1.5).

There was one patient receiving tezepelumab who stopped OCS use in DESTINATION and did achieve on-treatment clinical remission at week 52.

ACQ-6: Asthma Control Questionnaire-6; BD, bronchodilator; FEV_1: forced expiratory volume in 1 second; OCS: oral corticosteroid; Q4W: every 4 weeks.

FIGURE S3 The combinations of clinical remission criteria met by patients receiving tezepelumab 210 mg Q4W over a) weeks 0–52 and b) weeks >52–104.

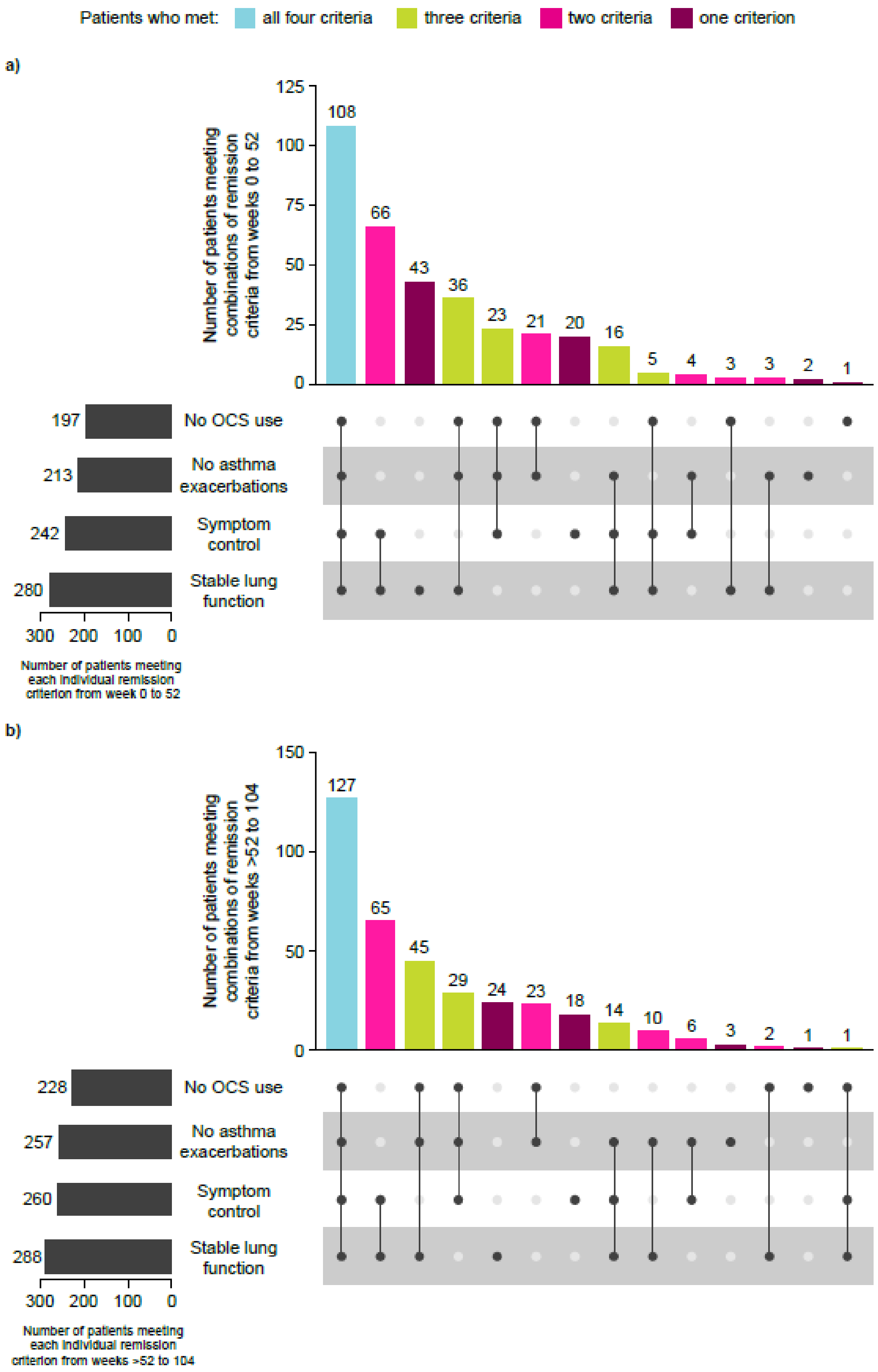

In this analysis, for patients who completed treatment with data missing at week 104, the next available off-treatment measurement was input at week 104 for the ACQ-6 and pre-BD FEV_1 criteria.

There was one patient receiving tezepelumab who stopped OCS use in DESTINATION and did achieve on-treatment clinical remission at week 52.

ACQ-6: Asthma Control Questionnaire-6; BD, bronchodilator; FEV_1: forced expiratory volume in 1 second; OCS: oral corticosteroid; Q4W: every 4 weeks.

FIGURE S4 The combinations of clinical remission criteria met by patients receiving placebo over a) weeks 0–52 and b) weeks >52–104.

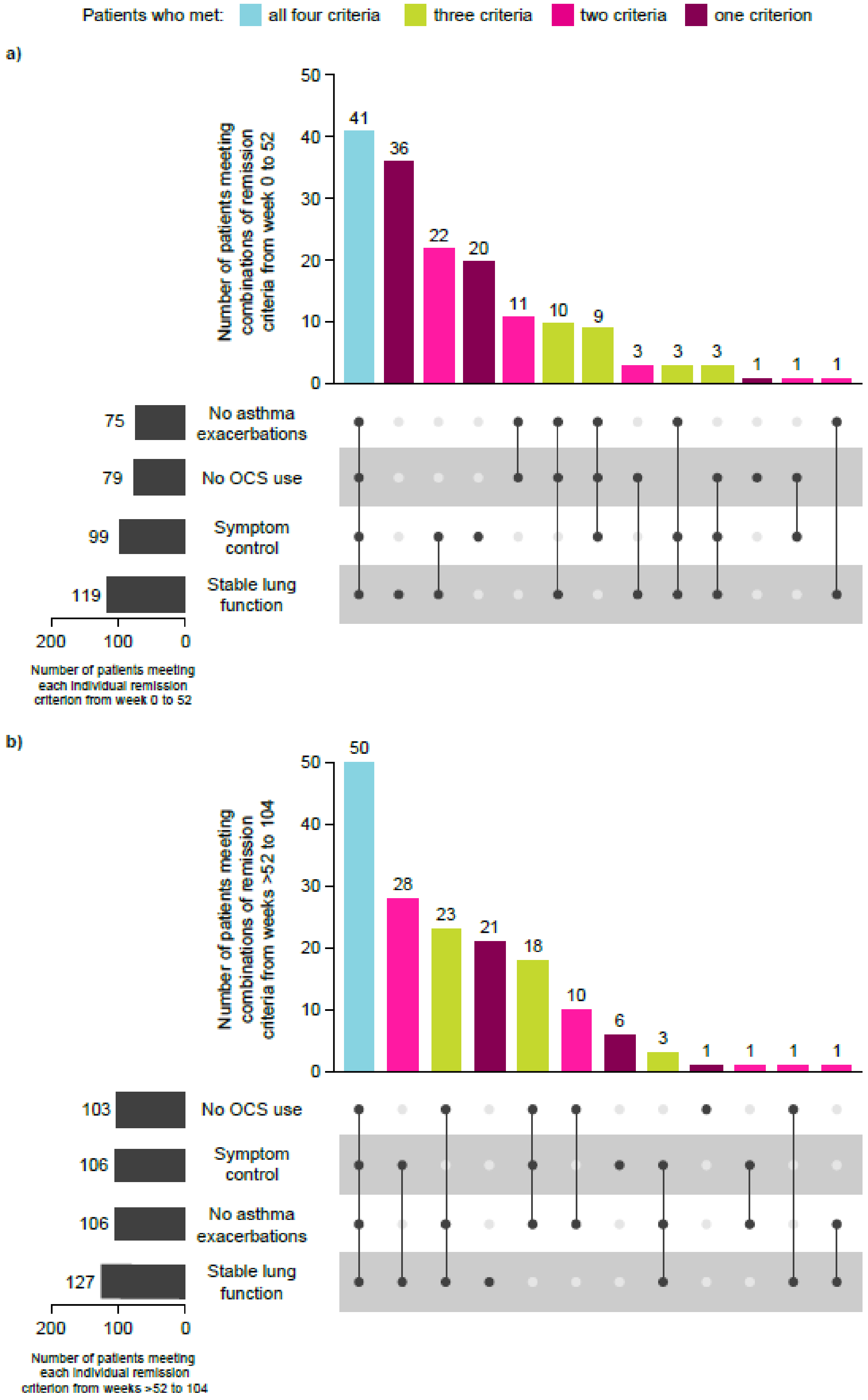

In this analysis, for patients who completed treatment with data missing at week 104, the next available off-treatment measurement was input at week 104 for the ACQ-6 and pre-BD FEV_1 criteria.

ACQ-6: Asthma Control Questionnaire-6; BD, bronchodilator; FEV_1: forced expiratory volume in 1 second; OCS: oral corticosteroid.

TABLE S1 Baseline demographics and clinical characteristics of complete responders and partial/non-responders from NAVIGATOR

Demographic/characteristic	Tezepelumab 210 mg Q4W (on-treatment, n2=431)[#]		Placebo (on-treatment, n2=420)[#]	
	Complete responders (n=199)	Partial/non-responders (n=232)	Complete responders (n=99)	Partial/non-responders (n=321)
**Age, years, mean (SD)**	48.3 (16.9)	52.1 (14.9)	46 (19.8)	50 (14.1)
**Female, n (%)**	119 (59.8)	151 (65.1)	58 (58.6)	207 (64.5)
**BMI, kg/m^2, mean (SD)**	28 (6.3)	29.5 (7.8)	27.9 (7.3)	28.8 (7.1)
**ICS dose group,[¶] n (%)**				
Medium	45 (22.6)	61 (26.3)	28 (28.3)	82 (25.5)
High	154 (77.4)	171 (73.7)	71 (71.7)	238 (74.1)
**Maintenance OCS use, n (%)**	18 (9.0)	19 (8.2)	3 (3.0)	28 (8.7)
**Pre-bronchodilator FEV$_1$, L, mean (SD)**	1.80 (0.69)	1.84 (0.74)	1.90 (0.77)	1.85 (0.70)
**Percent predicted pre-bronchodilator FEV$_1$, %, mean (SD)**	60.6 (17.5)	63.9 (17.8)	62.6 (18.2)	62.8 (17.6)
**FEV$_1$ reversibility, %, mean (SD)**	18.5 (17.7)	11.9 (12.4)	20.0 (16.9)	13.5 (14.4)
**Exacerbations in the 12 months before enrolment in NAVIGATOR, n (%)**				
2	109 (54.8)	144 (62.1)	66 (66.7)	193 (60.1)
>2	90 (45.2)	88 (37.9)	33 (33.3)	128 (39.9)
**FeNO level, ppb**				
Mean (SD)	47.7 (38.9)	36.0 (33.6)	44.5 (43.8)	45.6 (42.9)
Median (min, max)	35.0 (5.0, 213.0)	26.0 (5.0, 235.0)	27.0 (5.0, 231.0)	31.0 (5.0, 258.0)
**FeNO group, ppb, n (%)**				

<25	65 (32.8)	107 (46.9)	46 (46.5)	123 (38.6)
≥25 to <50	61 (30.8)	68 (29.8)	23 (23.2)	104 (32.6)
≥50	72 (36.4)	53 (23.2)	30 (30.3)	92 (28.8)
**BEC, cells/µL**				
Mean (SD)	406 (363)	255 (209)	402 (821)	339 (395)
Median (min, max)	340 (20, 3650)	200 (0, 1340)	290 (10, 8170)	240 (0, 4640)
**BEC group, cells/µL, n (%)**				
<150	35 (17.6)	76 (32.8)	24 (24.2)	87 (27.1)
150 to <300	53 (26.6)	89 (38.4)	28 (28.3)	108 (33.6)
<300	88 (44.2)	165 (71.1)	52 (52.5)	195 (60.7)
≥300	111 (55.8)	67 (28.9)	47 (47.5)	126 (39.3)
300 to <450	44 (22.1)	39 (16.8)	22 (22.2)	52 (16.2)
≥450	67 (33.7)	28 (12.1)	25 (25.3)	74 (23.1)
**Serum total IgE, IU/mL**				
Mean (SD)	514.4 (756.4)	461.4 (1048.9)	842.5 (1361.3)	556.3 (1053.8)
Median (min, max)	206.1 (1.5, 4357.4)	160.9 (1.5, 12 823.2)	236.9 (3.8, 7406.3)	177.3 (1.5, 8900.0)
**FEIA positive for any perennial aeroallergen,[+] n (%)**	116 (58.3)	156 (67.2)	70 (70.7)	203 (63.2)
**Nasal polyps, n (%)**	43 (21.6)	33 (14.2)	11 (11.1)	47 (14.6)

[#]On-treatment n2 denotes the number of patients in the treatment group who completed the on-treatment period and had non-missing on-treatment data available at week 52.

[¶]Medium-dose ICS: fluticasone propionate 500 µg/day or equivalent; high-dose ICS: fluticasone propionate >500 µg/day or equivalent; there was one patient in the placebo group who received fluticasone propionate <500 µg/day or equivalent.

[+]Positive for at least one perennial aeroallergen (cat dander, dog dander, cockroach, dust mite [*Dermatophagoides farinae, D. pteronyssinus*] and mould mix).

BEC: blood eosinophil count; BMI: body mass index; FEIA: fluorescence enzyme immunoassay; FeNO: fractional exhaled nitric oxide; FEV_1: forced expiratory volume in 1 second; ICS: inhaled corticosteroid; IgE: immunoglobulin E; OCS: oral corticosteroid; Q4W: every 4 weeks; SD: standard deviation.

TABLE S2 Baseline demographics and clinical characteristics for DESTINATION patients (originally enrolled in the NAVIGATOR study) who completed the on-treatment period and who received only tezepelumab 210 mg Q4W or only placebo in both studies.

Demographic/characteristic	Tezepelumab 210 mg Q4W (n=379)	Placebo (n=187)
**Age, years, mean (SD)**	49.5 (16.5)	48.3 (16.8)
**Female, n (%)**	236 (62.3)	118 (63.1)
**BMI, kg/m^2, mean (SD)**	29.0 (7.2)	28.8 (7.3)
**ICS dose group, n (%)**[#]		
Medium	93 (24.5)	52 (27.8)
High	286 (75.5)	134 (71.7)
**Maintenance OCS use, n (%)**	25 (6.6)	11 (5.9)
**Exacerbations in the 12 months before enrolment in NAVIGATOR, n (%)**		
2	235 (62.0)	120 (64.2)
>2	144 (38.0)	67 (35.8)
**Pre-bronchodilator FEV$_1$, L, mean (SD)**	1.84 (0.70)	1.83 (0.67)
**Percent predicted pre-bronchodilator FEV$_1$, %, mean (SD)**	62.4 (17.4)	62.2 (16.7)
**FeNO level, ppb, median (min, max)**	30.0 (5.0, 213.0)	26.0 (5.0, 258.0)
**BEC, cells/µL, median (min, max)**	250 (0, 3650)	230 (10, 1140)
**Serum total IgE, IU/mL, median (min, max)**	206.1 (1.5, 12 823.2)	172.3 (1.5, 7632.3)
**FEIA positive for any perennial aeroallergen,**[¶] **n (%)**	242 (63.9)	121 (64.7)
**Nasal polyps, n (%)**	72 (19.0)	23 (12.3)

[#]Medium-dose ICS: fluticasone propionate 500 µg/day or equivalent; high-dose ICS: fluticasone propionate >500 µg/day or equivalent; there was one patient in the placebo group who received fluticasone propionate <500 µg/day or equivalent.

[¶]Positive for at least one perennial aeroallergen (cat dander, dog dander, cockroach, dust mite [*Dermatophagoides farinae, D. pteronyssinus*] and mould mix).

BEC: blood eosinophil count; BMI: body mass index; FEIA: fluorescence enzyme immunoassay; FeNO: fractional exhaled nitric oxide; FEV$_1$: forced expiratory volume in 1

second; ICS: inhaled corticosteroid; IgE: immunoglobulin E; OCS: oral corticosteroid; Q4W: every 4 weeks; SD: standard deviation.

TABLE S3 Baseline demographics and clinical characteristics for patients receiving placebo who achieved and did not achieve clinical remission over weeks 0–24, >24–52 and >52–104.

	**Achieved remission**			**Did not achieve remission**		
	**Time point, weeks**					
**Demographic/characteristic**	**0–24 (n=57)**	**>24–52 (n=49)**	**>52–104[#] (n=50)**	**0–24 (n=126)**	**>24–52 (n=135)**	**>52–104[#] (n=132)**
**Age, years, mean (SD)**	47.2 (16.4)	45.1 (19.3)	42.9 (19.8)	49.0 (16.9)	49.8 (15.6)	50.8 (14.5)
**Female, n (%)**	36 (63.2)	31 (63.3)	29 (58.0)	79 (62.7)	86 (63.7)	86 (65.2)
**BMI, kg/m^2, mean (SD)**	28.4 (7.2)	28.2 (7.7)	27.7 (6.2)	29.0 (7.4)	29.2 (7.3)	29.4 (7.7)
**ICS dose group, n (%)[¶]**						
Medium	24 (42.1)	17 (34.7)	21 (42.0)	28 (22.2)	33 (24.4)	29 (22.0)
High	33 (57.9)	32 (65.3)	29 (58.0)	97 (77.0)	101 (74.8)	102 (77.3)
**Pre-bronchodilator FEV$_1$, L, mean (SD)**	1.94 (0.72)	1.94 (0.67)	1.94 (0.70)	1.80 (0.64)	1.80 (0.67)	1.77 (0.65)
**Percent predicted pre-bronchodilator FEV$_1$, %, mean (SD)**	63.8 (14.5)	65.0 (15.6)	64.0 (15.7)	61.7 (17.7)	61.2 (16.8)	60.8 (16.7)
**FEV$_1$ reversibility, %, mean (SD)**	16.3 (17.0)	17.0 (15.6)	16.5 (18.3)	14.0 (15.4)	14.2 (16.1)	14.7 (15.1)
**Age of asthma onset, years, mean (SD)**	22.1 (16.0)	20.5 (16.9)	17.7 (16.9)	26.7 (19.1)	27.2 (18.7)	28.4 (17.9)
**Duration of disease, years, mean (SD)**						
<20	11.0 (4.9)	10.9 (4.4)	11.0 (4.2)	10.0 (4.9)	10.0 (5.2)	9.9 (5.2)
≥20	35.6 (12.9)	34.4 (12.0)	36.7 (12.2)	35.4 (13.8)	35.8 (14.0)	35.0 (14.0)

**Duration of disease, years, n (%)**						
<20	25 (43.9)	21 (42.9)	23 (46.0)	66 (52.4)	70 (51.9)	67 (50.8)
≥20	32 (56.1)	28 (57.1)	27 (54.0)	60 (47.6)	65 (48.1)	65 (49.2)
**Exacerbations in the past 12 months, n (%)**						
2	44 (77.2)	37 (75.5)	39 (78.0)	75 (59.5)	81 (60.0)	79 (59.8)
>2	13 (22.8)	12 (24.5)	11 (22.0)	51 (40.5)	54 (40.0)	53 (40.2)
**FeNO level, ppb**						
Mean (SD)	32.4 (24.8)	33.1 (29.9)	39.8 (45.8)	46.8 (49.6)	45.2 (47.3)	43.3 (43.1)
Median (min, max)	23.0 (9.0, 105.0)	23.0 (8.0, 132.0)	24.0 (8.0, 231.0)	27.0 (5.0, 258.0)	27.0 (5.0, 258.0)	27.0 (5.0, 258.0)
**FeNO group, ppb, n (%)**						
<25	30 (52.6)	27 (55.1)	25 (50.0)	57 (46.0)	61 (45.9)	61 (46.6)
≥25 to <50	15 (26.3)	13 (26.5)	16 (32.0)	31 (25.0)	33 (24.8)	31 (23.7)
≥50	12 (21.1)	9 (18.4)	9 (18.0)	36 (29.0)	39 (29.3)	39 (29.8)
**BEC, cells/µL**						
Mean (SD)	273 (185)	282 (181)	274 (197)	289 (225)	287 (224)	289 (218)
Median (min, max)	230 (10, 980)	240 (10, 980)	225 (20, 980)	225 (40, 1140)	210 (40, 1140)	235 (10, 1140)
**BEC group, cells/µL, n (%)**						
<150	13 (22.8)	11 (22.4)	13 (26.0)	39 (31.0)	41 (30.4)	38 (28.8)
150 to <300	25 (43.9)	17 (34.7)	19 (38.0)	39 (31.0)	45 (33.3)	44 (33.3)
<300	38 (66.7)	28 (57.1)	32 (64.0)	78 (61.9)	86 (63.7)	82 (62.1)
≥300	19 (33.3)	21 (42.9)	18 (36.0)	48 (38.1)	49 (36.3)	50 (37.9)
300 to <450	9 (15.8)	12 (24.5)	11 (22.0)	24 (19.0)	24 (17.8)	24 (18.2)
≥450	10 (17.5)	9 (18.4)	7 (14.0)	24 (19.0)	25 (18.5)	26 (19.7)
**Serum total IgE, IU/mL**						
Mean (SD)	388.6 (573.0)	438.2 (728.0)	649.2 (940.2)	591.4 (1058.2)	586.0 (1022.9)	515.5 (969.2)

Median (min, max)	146.8 (3.2, 2838.4)	131.3 (15.0, 3200.4)	271.3 (1.5, 4746.8)	193.5 (1.5, 7632.3)	203.6 (1.5, 7632.3)	165.5 (1.5, 7632.3)
**FEIA positive for any perennial aeroallergen,[+] n (%)**	40 (70.2)	35 (71.4)	37 (74.0)	79 (62.7)	83 (61.5)	80 (60.6)
**Nasal polyps, n (%)**	6 (10.5)	4 (8.2)	5 (10.0)	16 (12.7)	19 (14.1)	18 (13.6)

[#]In this analysis, for patients who completed treatment with data missing at week 104, the next available off-treatment measurement was input at week 104 for the ACQ-6 and pre-BD FEV_1 criteria.

[¶]Medium-dose ICS: fluticasone propionate 500 µg/day or equivalent; high-dose ICS: fluticasone propionate >500 µg/day or equivalent; there was one patient in the placebo group who received fluticasone propionate <500 µg/day or equivalent.

[+]Positive for at least one perennial aeroallergen (cat dander, dog dander, cockroach, dust mite [*Dermatophagoides farinae, D. pteronyssinus*] and mould mix).

ACQ-6: Asthma Control Questionnaire-6; BD, bronchodilator; BEC: blood eosinophil count; BMI: body mass index; FEIA: fluorescence enzyme immunoassay; FeNO: fractional exhaled nitric oxide; FEV_1: forced expiratory volume in 1 second; ICS: inhaled corticosteroid; IgE: immunoglobulin E; OCS: oral corticosteroid; SD: standard deviation.
