# Supplementary material for: Clinical response and on-treatment clinical remission with tezepelumab in a broad population of patients with severe, uncontrolled asthma: results over 2 years from the NAVIGATOR and DESTINATION studies
Source: Eur Respir J. 2024 Dec 5;64(6):2400316. doi: 10.1183/13993003.00316-2024 (PMC11618813; doi:10.1183/13993003.00316-2024)
Supplement: Supplementary file 2 [file ERJ-00316-2024.Shareable.pdf]

SHAREABLE PDF

EUROPEAN RESPIRATORY JOURNAL
ORIGINAL RESEARCH ARTICLE

Clinical response and on-treatment clinical remission with tezepelumab in a broad population of patients with severe, uncontrolled asthma: results over 2 years from the NAVIGATOR and DESTINATION studies

Michael E. Wechsler, Guy Brusselle, J. Christian Virchow, Arnaud Bourdin, Konstantinos Kostikas, Jean-Pierre Llanos, Stephanie L. Roseti, Christopher S. Ambrose, Gillian Hunter, David J. Jackson, Mario Castro, Njira Lugogo, Ian D. Pavord, Neil Martin and Christopher E. Brightling

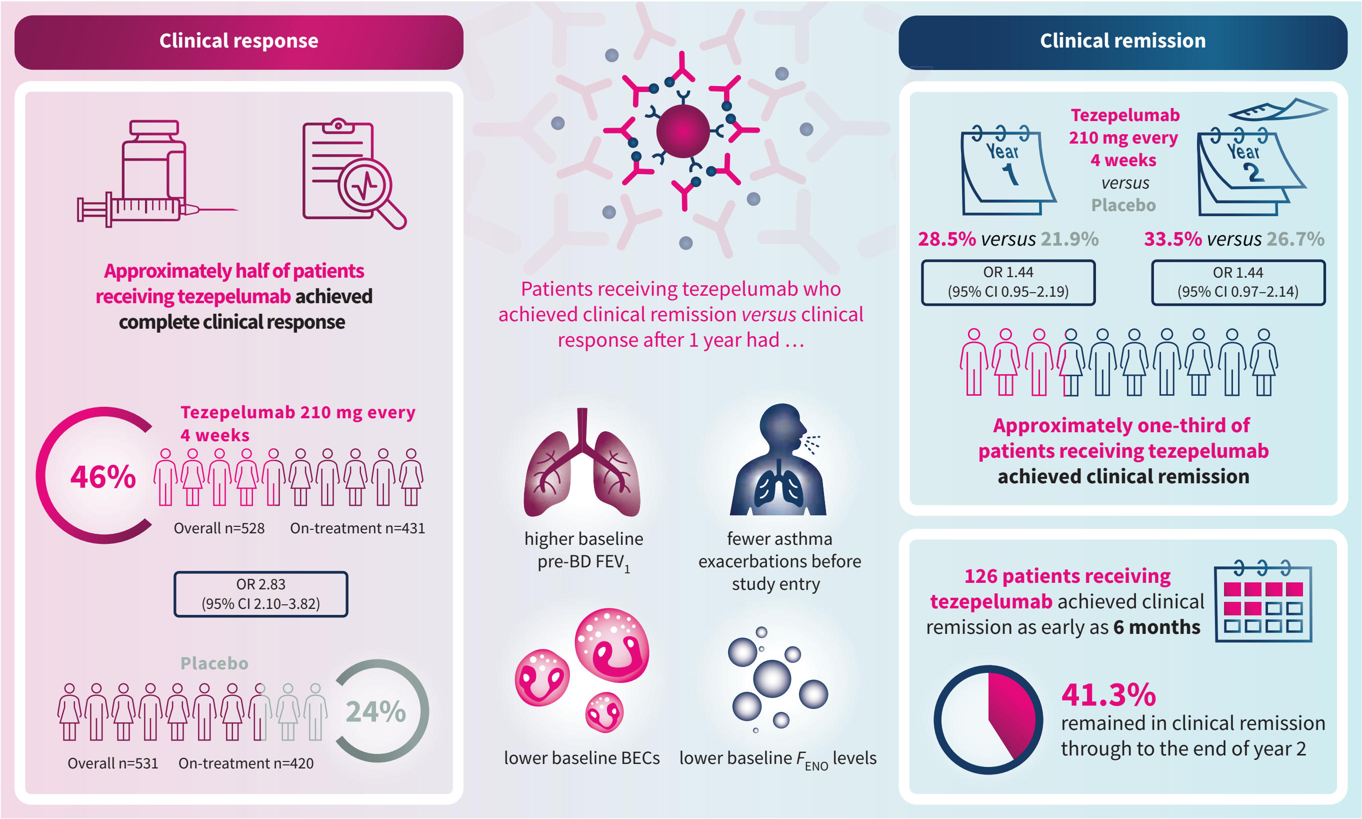

GRAPHICAL ABSTRACT Tezepelumab treatment was associated with an increased likelihood of achieving a complete clinical response and on-treatment clinical remission *versus* placebo in patients with severe, uncontrolled asthma. Clinical response data are taken from the NAVIGATOR phase 3 trial (52 weeks). Clinical remission data are taken from the DESTINATION phase 3 trial (104 weeks). BD: bronchodilator; BEC: blood eosinophil count; F_{ENO}: fractional exhaled nitric oxide; FEV_1: forced expiratory volume in 1 s.

Link to published version: https://doi.org/10.1183/13993003.00316-2024

EUROPEAN RESPIRATORY JOURNAL
ORIGINAL RESEARCH ARTICLE
M.E. WECHSLER ET AL.

SHAREABLE PDF

Clinical response and on-treatment clinical remission with tezepelumab in a broad population of patients with severe, uncontrolled asthma: results over 2 years from the NAVIGATOR and DESTINATION studies

Michael E. Wechsler[1], Guy Brusselle[2], J. Christian Virchow[3], Arnaud Bourdin[4], Konstantinos Kostikas[5], Jean-Pierre Llanos[6], Stephanie L. Roseti[7], Christopher S. Ambrose[8], Gillian Hunter[9], David J. Jackson[10,11], Mario Castro[12], Njira Lugogo[13], Ian D. Pavord[14], Neil Martin[15,16] and Christopher E. Brightling[15]

[1]Division of Pulmonary, Critical Care and Sleep Medicine, National Jewish Health, Denver, CO, USA. [2]Department of Respiratory Medicine, Ghent University Hospital, Ghent, Belgium. [3]Department of Pneumology and Department of Intensive Care Medicine, University of Rostock, Rostock, Germany. [4]PhyMedExp, University of Montpellier, CNRS, INSERM, CHU Montpellier, Montpellier, France. [5]Respiratory Medicine Department, University of Ioannina, Ioannina, Greece. [6]Global Medical Affairs, Amgen, Thousand Oaks, CA, USA. [7]Late-stage Development, Respiratory and Immunology, BioPharmaceuticals R&D, AstraZeneca, Gaithersburg, MD, USA. [8]Respiratory and Immunology, BioPharmaceuticals Medical, AstraZeneca, Gaithersburg, MD, USA. [9]Biometrics, Late-stage Development, Respiratory and Immunology, BioPharmaceuticals R&D, AstraZeneca, Cambridge, UK. [10]Guy's Severe Asthma Centre, Guy's and St Thomas' NHS Foundation Trust, London, UK. [11]School of Immunology and Microbial Sciences, King's College London, London, UK. [12]Division of Pulmonary, Critical Care and Sleep Medicine, University of Kansas School of Medicine, Kansas City, KS, USA. [13]Department of Medicine, Division of Pulmonary and Critical Care Medicine, University of Michigan, Ann Arbor, MI, USA. [14]Respiratory Medicine, National Institute for Health and Care Research, Oxford Biomedical Research Centre, Nuffield Department of Medicine, University of Oxford, Oxford, UK. [15]Institute for Lung Health, National Institute for Health and Care Research, Leicester Biomedical Research Centre, University of Leicester, Leicester, UK. [16]Respiratory and Immunology, BioPharmaceuticals Medical, AstraZeneca, Cambridge, UK.

Corresponding author: Christopher E. Brightling (ceb17@leicester.ac.uk)

Check for updates

Shareable abstract (@ERSpublications)
Tezepelumab treatment was associated with an increased likelihood of achieving a complete clinical response over 52 weeks as well as achieving on-treatment clinical remission over 2 years in patients with severe, uncontrolled asthma https://bit.ly/3TiAkA7

Cite this article as: Wechsler ME, Brusselle G, Virchow JC, *et al*. Clinical response and on-treatment clinical remission with tezepelumab in a broad population of patients with severe, uncontrolled asthma: results over 2 years from the NAVIGATOR and DESTINATION studies. *Eur Respir J* 2024; 64: 2400316 [DOI: 10.1183/13993003.00316-2024].

This extracted version can be shared freely online.

Copyright ©The authors 2024.

This version is distributed under the terms of the Creative Commons Attribution Licence 4.0.

This article has an editorial commentary: https://doi.org/10.1183/13993003.01908-2024

Received: 15 Feb 2024
Accepted: 23 Aug 2024

Abstract

Background In asthma, clinical response is characterised by disease improvement with treatment, whereas clinical remission is characterised by long-term disease stabilisation with or without ongoing treatment. The proportions of patients receiving tezepelumab who responded to treatment and who achieved on-treatment clinical remission were assessed in the NAVIGATOR (ClinicalTrials.gov identifier NCT03347279) and DESTINATION (ClinicalTrials.gov identifier NCT03706079) studies of severe, uncontrolled asthma.

Methods NAVIGATOR and DESTINATION were phase 3, randomised, double-blind, placebo-controlled studies; DESTINATION was an extension of NAVIGATOR. Complete clinical response was defined as achieving all of the following: ⩾50% reduction in exacerbations *versus* the previous year, improvements in pre-bronchodilator (BD) forced expiratory volume in 1 s (FEV_1) of ⩾100 mL or ⩾5%, improvements in Asthma Control Questionnaire (ACQ)-6 score of ⩾0.5 and physician's assessment of asthma improvement. On-treatment clinical remission was defined as an ACQ-6 total score ⩽1.5, stable lung function (pre-BD FEV_1 >95% of baseline) and no exacerbations or use of oral corticosteroids during the time periods assessed.

Results Higher proportions of tezepelumab than placebo recipients achieved complete clinical response over weeks 0–52 (46% *versus* 24%; OR 2.83, 95% CI 2.10–3.82) and on-treatment clinical remission over

weeks 0–52 (28.5% *versus* 21.9%; OR 1.44, 95% CI 0.95–2.19) and weeks >52–104 (33.5% *versus* 26.7%; OR 1.44, 95% CI 0.97–2.14). Tezepelumab recipients who achieved on-treatment clinical remission *versus* complete clinical response at week 52 had better preserved lung function and lower inflammatory biomarker levels at baseline, and fewer exacerbations in the 12 months before the study.

Conclusions Among patients with severe, uncontrolled asthma, tezepelumab treatment was associated with an increased likelihood of achieving complete clinical response and on-treatment clinical remission compared with placebo. Both are clinically important outcomes, but may be driven by different patient characteristics.
